# Malaria Parasitaemia and the use of insecticide-treated nets (INTs) for malaria control amongst under-5 year old children in Calabar, Nigeria

**DOI:** 10.1186/s12879-016-1459-5

**Published:** 2016-04-14

**Authors:** Anthony Achizie Iwuafor, Chukwudi Charles Egwuatu, Agwu Ulu Nnachi, Ita Okokon Ita, Godwin Ibitham Ogban, Comfort Nneka Akujobi, Tenny Obiageli Egwuatu

**Affiliations:** Department of Medical Microbiology and Parasitology, College of Medical Sciences, University of Calabar, Calabar, Nigeria; Department of Medical Microbiology and Parasitology, Faculty of Medicine, Nnamdi Azikiwe University, Nnewi Campus, Nigeria; Department of Immunology, Faculty of Medicine, Nnamdi Azikiwe University, Nnewi Campus, Nigeria; Department of Medical Microbiology and Parasitology, Faculty of Science, University of Lagos, Akoka, Lagos Nigeria

**Keywords:** Caregiver, Insecticide-treated net (ITNs), Parasitaemia, Plasmodium falciparum, Nigeria

## Abstract

**Background:**

Malaria remains a major cause of febrile illness in Nigeria and interventions to reduce malaria burden in Nigeria focus on the use of insecticide-treated nets. This study determined the prevalence of malaria parasitaemia and the use of insecticide-treated nets (ITNs) for the control of malaria amongst under-five year old children in Calabar, Nigeria.

**Methods:**

A total of 270 under-5 year old children were recruited and structured questionnaires were used to obtain information on the background characteristics of the respondents from their caregivers. Capillary blood samples were collected from each of the patients through finger-pricking and tested for malaria parasites by Rapid Diagnostic Test and microscopy.

**Results:**

An overall parasitaemia prevalence of 32.2 % (by Rapid diagnostic test kit [RDT]) and 40.1 % (by microscopy) were obtained in this study. Forty-six (45.5 %) of the febrile patients had malaria parasitaemia (by RDT) or 41 (59.4 %) by microscopy. One hundred and fifty (55.6 %) of the caregivers acknowledged the use of nets on doors and windows for malaria prevention and control. One hundred and thirty-nine (51.5 %) mentioned sleeping under mosquito net while 138 (51.1 %) acknowledged the use of insecticide sprays. Although 191 (71.5 %) of the households possessed at least one mosquito net, only 25.4 % of the under-5 children slept under any net the night before the survey. No statistically significant reduction in malaria parasitaemia was observed with the use of mosquito nets among the under-5 children. Almost all the respondents (97.8 %) identified mosquito bite as the cause of malaria. Fever was identified by the majority of the respondents (92.2 %) as the most common symptom of malaria.

**Conclusions:**

The findings of the study showed high prevalence of parasitaemia and that fever was significantly associated with malaria parasitaemia. Mosquito net utilization among the under-fives was low despite high net ownership rate by households. Therefore, for effective control of malaria, public health education should focus on enlightening the caregivers on signs/symptoms of both uncomplicated and complicated malaria as well as encourage the use of ITNs especially among the under-fives.

**Electronic supplementary material:**

The online version of this article (doi:10.1186/s12879-016-1459-5) contains supplementary material, which is available to authorized users.

## Background

Febrile illness is the most common and important component of malaria syndrome in sub-Saharan Africa [1]. Malaria remains one of the most widespread diseases affecting human race in tropical and sub-tropical regions of the world [2]. According to World malaria report, an estimated 3.3 billion people were at risk of malaria in 2010. Of this total, 1.2 billion were at high risk (>1 case per 1000 population), 47 % of them were living in Africa while 37 % came from South-East Asia [3]. There were 216 million episodes of malaria in 2010, and approximately 81 % or 174 million cases were in African Region. There were an estimated 655,000 malaria deaths in 2010 of which 91.0 % occurred in the African Region, and 86.0 % of the deaths involved children under the age of five years [3]. Malaria is caused by five different species of *Plasmodium* parasites and transmitted by female Anopheles mosquito [4]. In Nigeria, *Plasmodium falciparum* is the most dominant malaria parasites (>95.0 %), with *P. ovale* and *P. malariae* being responsible for the remainder. Dominant vector species are *Anopheles gambiaes. l*. and the *Anopheles funestus* group with some other groups playing a minor role [5].

Reductions in malaria disease burden, as documented in the recent World Malaria Reports [6, 7], have coincided with the massive scale-up of malaria prevention measures, of which vector control was the predominant component, particularly in sub-Saharan Africa. The core malaria vector control interventions are insecticide-treated nets (ITNs) and indoor residual spraying (IRS), both of which deploy insecticides to kill malaria-transmitting mosquitoes [8].

The Federal Government of Nigeria, therefore, developed the National Malaria Control Strategic Plan 2000–2005, 2006–2010 which due to limited resources was targeted on the vulnerable groups of pregnant women and children under 5 years old. The interventions focused on the use of Long Lasting Insecticidal Nets [LLINs]/Insecticide-Treated Nets [ITNs] and Artemisinin Combination Therapy (ACT). The distribution of LLINs was integrated with Ante Natal Care, immunization as well as stand-alone campaigns [9]. Also, other organizations which include Faith-based organizations, Non-governmental organizations, and World Bank, with the goal of achieving universal access for the at-risk population of under 5 year old and pregnant women have been involved in free distribution of LLINs/ITNs [9]. Use of ITNs has been proven to be very effective in reducing malaria and malaria-associated morbidity among preschool children [10].

The role caregivers, especially mothers, play in attending to their febrile child is very important in reducing morbidity and mortality due to malaria. This is most important where the place is considered high risk for malaria, i.e., if > 5 % of fevers among children is caused by malaria. For example, a child with fever in a high-risk area who does not appear to have any other underlying reason for the fever, e.g., measles on physical evaluation should be considered as having malaria. Such a child should receive anti-malarial drugs. This is the WHO programme guidelines for Integrated Management of Childhood Illness [IMCI], used in resource limited settings to evaluate and treat children [11].

Despite the evidence-based benefits of sleeping under ITNs, and the efforts made by the Federal Government and Non-governmental Organizations to tame the public health scourge of malaria in Nigeria, some geopolitical zones of the country still record low average number of ITNs ownership/usage per household [12]. Different reasons have been advanced for poor ownership and usage of ITNs, by caregivers. Hence, this study investigated the prevalence of malaria infection and the use of insecticide-treated nets (ITNs) for malaria control among under-five children in Calabar, Nigeria.

## Methods

### Study design/setting

The study is descriptive and cross-sectional in design. It was carried out from November, 2012 to December, 2013 to determine malaria parasitaemia and the perception and practices of care-givers of under-five children on the use of ITNs amongst the under-five children. It was carried out in the University of Calabar Teaching Hospital, Calabar which is a second generation Teaching hospital in the country, Nigeria. The hospital currently has over 600 beds distributed between the three annexes and renders services in specialized areas in medicine such as paediatric surgery, haemodialysis, neuro-surgery, ophthalmologic surgery and maternal health.

### Participants

The study target population consisted of women/men aged 15 – 50 years who had the responsibility of taking care of at least one Under-five year old child. A total of 270 under-5 children who came to the hospital as outpatients with their care-givers were recruited in the study.

### Ethical considerations

Approval was obtained from the Research and Ethics Committee of the University of Calabar Teaching Hospital, Calabar, Nigeria. Informed consent was also obtained from the patients’ relatives. Those who declined consent were excluded from the study.

### Sample size

Single population proportion formula (N = Z^2^ pq/d^2^) was used to determine the sample size assuming the ITN usage rate among under five children in the South-South zone of Nigeria to be 20.0 % [12] at 95 % confidence interval, 5 % marginal error, and 10 % non-response rate. This gave a sample size of 270 under five children.

### Data collection

#### Data collection procedures

A convenient sampling method was employed to select the calculated sample size of under-five caregivers/Under-five year old respondents. As many of the respondents who gave consent on each of their clinic day was enrolled into the study during the study period until the sample size was complete.

Each caregiver who attended clinic with their child (ren) was interviewed by trained interviewers using structured questionnaires (Additional file 1) adapted from NPC-NMCP Nigeria Malaria Indicator Survey [12]. The selection of the interviewers was based on the respondent’s ability to understand English and the local language (Efik/Ibibio language) because where necessary, the interviewer had to interpret the questionnaire in the language of the respondents. The questionnaire was pre-tested to check for comprehensibility of the questions as well as the procedures for conducting the interviews. The questionnaire elicited information on: background characteristics of respondents, knowledge of malaria symptoms, causes of malaria, ways to avoid malaria and knowledge of prompt treatment of children with fever. Other information that was captured by the questionnaire included: household possession and use of mosquito nets, source and cost of mosquito nets, reasons for non-use of nets and febrile illness-associated mortalities one year prior to survey (Additional file 1).

#### Sample collection and processing

Fresh capillary blood samples were collected aseptically from the recruited under-5 children using finger-pricking method as documented by Cheesbrough [13]. The sample was processed immediately using Paracheck Pf® Rapid diagnostic Test kit (Orchid Biomedical Systems, India). In this, a drop of the whole fresh capillary blood was applied to the sample well ‘A’ and immediately, the specimen was blotted. Six drops of the clearing buffer was then made into well ‘B’ and the setup was allowed to stand undisturbed for 15 min. At the end of 15 min, results were read as follows: if only one pink-coloured band appeared in the control window, test was interpreted as negative. In addition to the control band, if a distinct pink coloured band also appeared in the test window, test was interpreted as positive. Test was considered invalid/inconclusive if no bands appeared on the device. In that case, test was repeated with new device ensuring that the test procedure was followed accurately. One hundred and sixty seven (167) of the rapid diagnostic tests carried out were correlated with microscopy. In this, thick blood smears and thin blood films were made in the field (clinics) and transported to the Paediatrics side-laboratory, where it was stained using 10 % Giemsa for 10 min by standard techniques [13]. Each slide was examined for the presence or absence of malaria parasites. Each slide was declared positive if at least one parasite was found per 100 high power fields; else, it was reported as negative. In this study, finding of at least one malaria parasite per 100 high power fields is considered positive parasitaemia. Quality of the microscopic slides was ensured by cross checking both the negative and positive slides by other trained Microscopist. Fever was measured with clinical thermometer and was defined as an auxiliary temperature of ≥ 37.5 °C.

### Statistical analyses

Statistical analysis was performed using Statistical Package for Social Sciences (SPSS) software (version 20.0, SPSS Inc., Chicago, IL., USA). Continuous variables were presented as the mean ± standard deviation. Categorical variables were presented as actual numbers and percentages in table forms, or figures. All categorical variables were compared using Pearson’s Chi-square test or Fisher’s exact test. *P*-values < 0.05 were considered significant for all tests. The outcome variables considered were ownership of ITNs, Use of ITNs a night prior to interview and the effect of ITN usage on malaria parasitaemia amongst under-five year children. Multivariate logistic regression analysis was employed to explore the impact of independent variables such as the child’s age, care-givers age, care-givers tribe and care-givers level of education on the outcome variables. The regression model used predicted the logit, which is the natural log of the odds of having made one or the other decision:

ln (Odds) = ln (Ý/1-Ý) = b_0_ + b_1_X_1_ + b_2_X_2_ + … + b_p_X_p_, where Ý is the predicted probability of the event which is coded with ‶0″ (Did not own ITN, Did not sleep under ITN and Negative Parasitaemia) rather than “1” (Own at least one ITN, Slept under an ITN, and Positive Parasitaemia). “1-Ý” is the predicted probability of the other decision and X_1_ through X_p_are distinct independent (predictor) variables and b_0_ through b_p_are the regression coefficients. The “-2 Log Likelihood” statistics and Hosmer-Lemeshow test were used to show how well the model predicts the decisions. Two tailed *P*-values was reported, odds ratios and 95 % Confidence interval was used to estimate the association between dependent (outcome) variables and independent variables.

## Results

### Baseline characteristics

A summary of the baseline characteristics of the respondents is given in Table 1. A total of 270 care-givers (all female) participated in the study. The mean age of the participants was 29.7 ± 5.6 standard deviation. Seventy seven percent of them fell into age-group of 26–35 years. The mean age-group (months) of the under-5 year old children whose care-givers were interviewed was 25.5 ± 17.3 standard deviation. The infants constituted 21.8 % of the children. The Efik tribe (53 %), followed by Igbo (19.5 %), were the most populous tribe in the study. Most of the respondents had tertiary education (58.9 %), only three (1.1 %) did not have any formal education whatsoever. One hundred (40.3 %) of the care-givers were civil servants,87 (35.1 %) were self-employed while 25 (10.1 %) were house-wives.

**Table 1:** Baseline characteristics of the respondents

Characteristics	% Frequency/Mean (SD)	Total
Care-givers’ age (years)		
18-25	13.2	35
26-35	77.1	205
36 & above	9.8	26
Total	100	266
Mean age (±SD)	29.7 (±5.6)	
Child age (months)		
1-11	21.8	52
12-35	41.6	99
36-59	36.6	87
Total	100	238
Mean age (±SD)	25.5 (±17.3)	
Care-givers’ tribe		
Efik	53	141
Northern cross river	9.8	26
Igbo	19.5	52
Hausa	4.9	13
Yoruba	2.3	6
Others	10.5	28
Total	100	266
Care-givers’ education		
None	1.1	3
Primary	3.7	10
Secondary	36.3	98
Tertiary	58.9	159
Total	100	270
Care-givers’ occupation		
Civil service	40.3	100
Self employed	35.1	87
House wife	10.1	25
Others	14.5	36
Total	100	248

### ITNs ownership and usage

Table 2 shows a summary of ITN-associated questions and answers. The minimum and maximum numbers of ITNs owned by any household were 1 and 5 respectively, with mean, standard deviation of 2.4 and ±1.8. More than two-thirds (191/267; 71.5 %) of the care-givers had at least one ITN per household. Of the number that had at least one ITN, one hundred and seventy eight (93.2 %) of them obtained the nets free of charge, only 13 (6.8 %) procured theirs via purchasing.

**Table 2:** Descriptive analysis of ownership and use of insecticide-treated nets, and malaria prevention methods

Characteristics	% Frequency/Mean (SD)	Total
Number of nets per household	2.4 (±1.8)	
Household ownership of ITNs		
Yes	71.5	191
No	28.5	76
Total	100	267
How ITN was acquired		
Given free of charge	93.2	178
Bought	6.8	13
Total	100	191
Where ITNs were obtained		
Primary health centre	67.5	129
NGOs	15.2	29
Government hospitals	7.3	14
Shop/Supermarket	3.7	7
Church/Mosque	3.1	6
Pharmacy	2.1	4
Patent medicine store	1.0	2
Total	100	191
How long ago was the ITN obtained?		
<1Month	1.6	3
2-12 Months	48.1	90
13-24 Months	42.8	80
>24 Months	7.5	14
Total	100	187
When you got the Net, was it treated?		
Yes	93.7	179
No	1.6	3
Not sure	4.7	9
Total	100	191
Since you got the net, have you ever treated it?		
Yes	11	21
No	89	169
Total	100	190
Did any child sleep under the net the previous night?		
Yes	35.6	68
No	64.4	123
Total	100	191

Majority of the households (129/191; 67.5 %) that owned at least one net got them from the Primary health centre closest to them, twenty-nine (15.2 %) of them got theirs from Non-Governmental Organizations. Only 2 (1.0 %) got theirs from the Patent medicine store. About half the population of the respondents obtained their nets within 2–12 Months prior to the study, only 14 (7.5 %) got their nets more than 2 years prior to the study. Almost all the nets (93.7 %) were already-treated nets by the time they were procured. Twenty-one (11.0 %) care-givers admitted a secondary treatment of their nets by themselves after procurement.

**Fig. 1:**
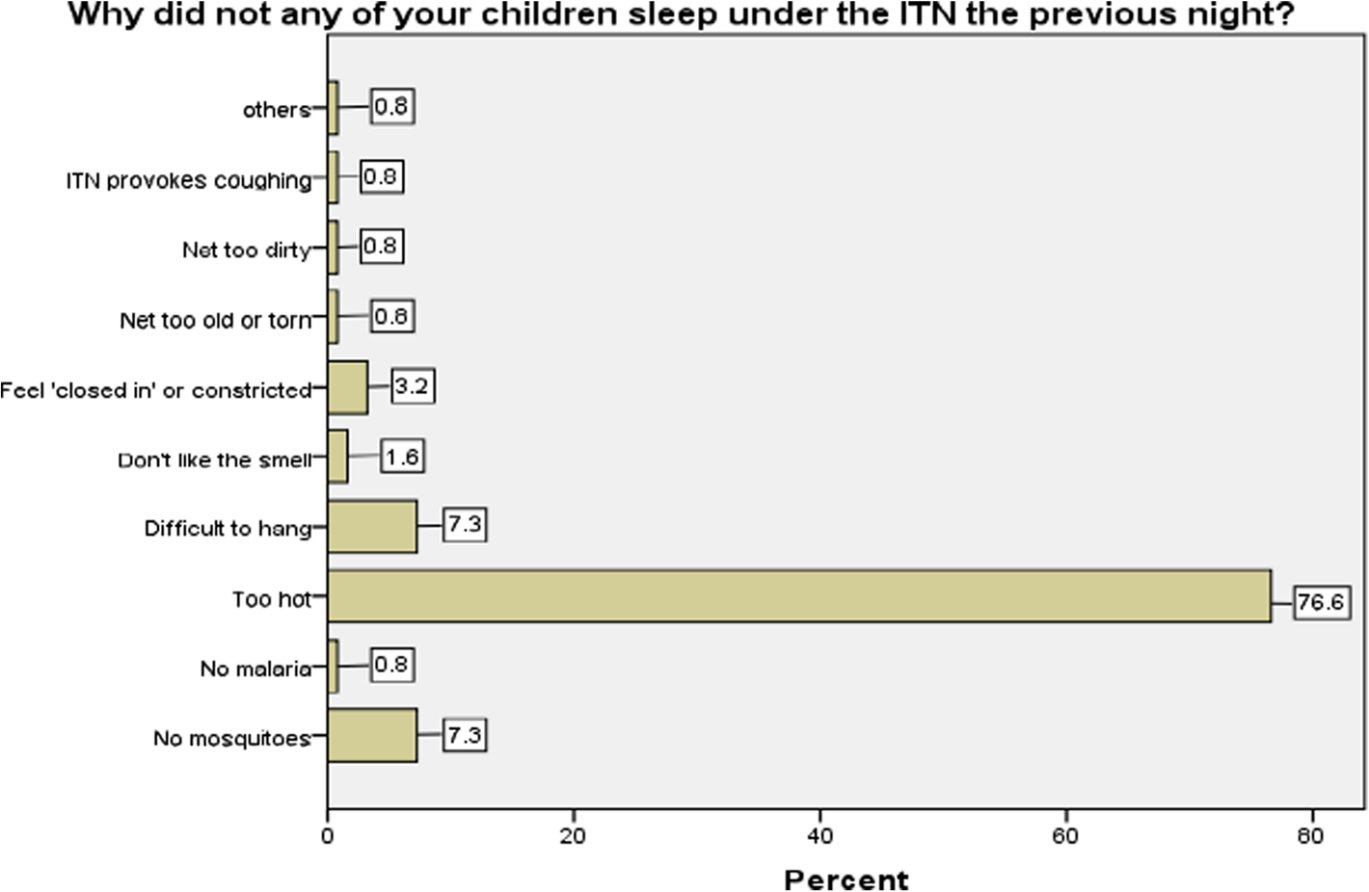
Reasons for not sleeping under ITN, the night prior to study

Sixty-eight (25.4 %) of the children studied were reported to have slept under any net the night before the survey. Amongst thosewho had nets (191), only 68 (35.6 %) had at least one under-5 year old child who slept under the net the night before the study. Those care-givers’ household in which no child slept under a net the night before this study gave different reasons for not sleeping under the net. Some of the reasons included: ‘weather was too hot’ (77.2 %), ‘difficulty at hanging the net’ (7.3 %), ‘there were no mosquitoes’ (7.3 %) (Fig. 1).

Reasons given for not having at least one ITN included: ‘nets not available (65.3 %), ‘don’t like to use nets’ (13.9 %), and ‘there is no mosquito’ (12.5 %).

### Caring for a febrile child

**Fig. 2:**
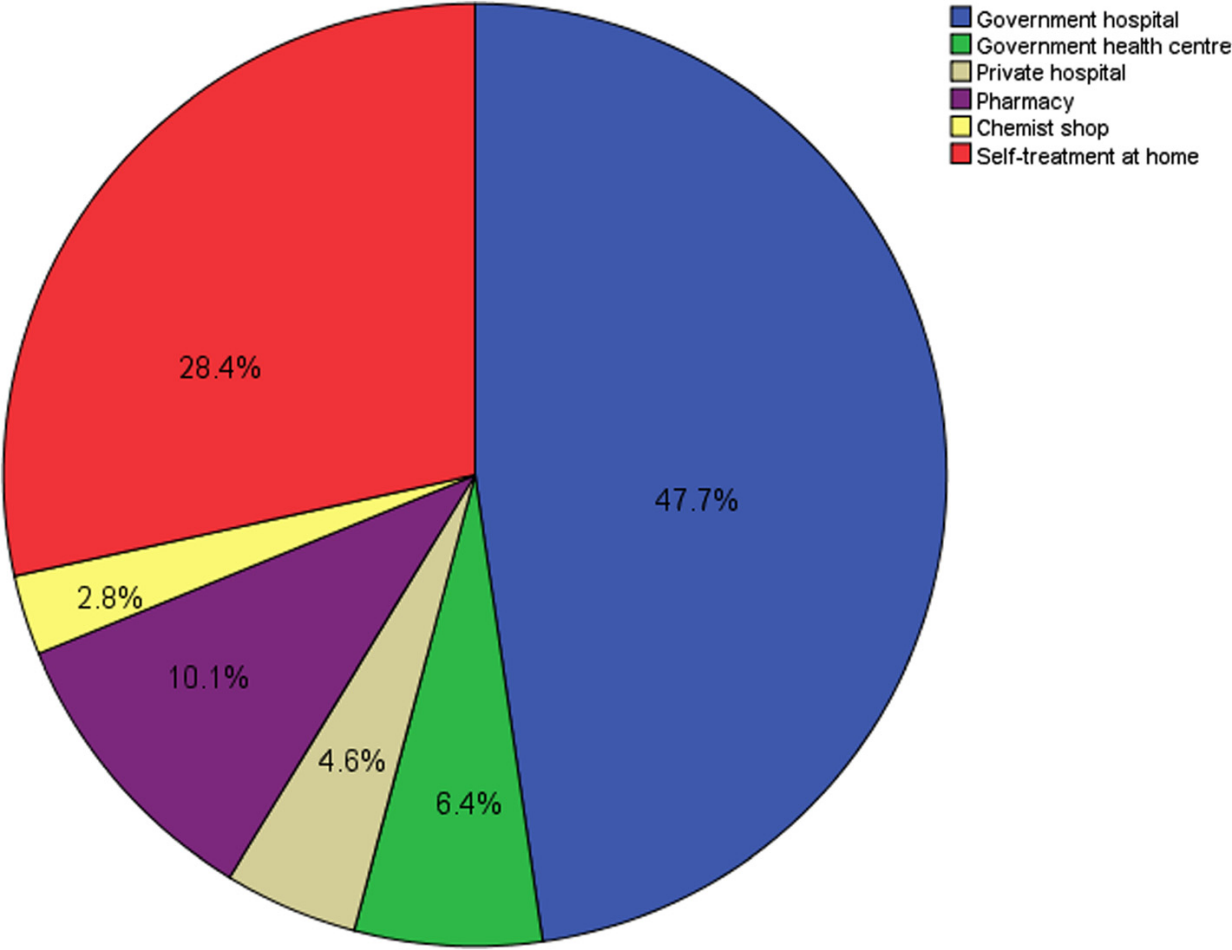
The first treatment modalities embarked upon by the Care-givers on instances of febrile illness

Figure 2 shows what the care-givers do when any under-5 year old child under them develops fever. This question applied only to those who admitted that any of the children under their care developed fever within two weeks prior to the study. Fifty-two (48 %) would take the child to any government hospital nearby, 31 (28.4 %) would administer ‘self-treatment’, while 11 (10.1 %) would consult a pharmacist. Other treatment modalities included taking the child to government health centre 7 (6.9 %), Private hospital 5 (4.9 %), and Chemist shop 3 (2.9 %).

### Malaria parasitaemia

Two hundred and seventy (270) patients were recruited, RDT tests were carried out on 258 patients and 32.2 % (83/258) had positive parasitaemia. On the hand, one hundred and sixty seven (167) were tested using slide microscopic method, 40.1 % (67/167) had positive slide (parasitaemia).

Two hundred and sixty-four (97.8 %) care-givers correctly identified mosquito bite as the cause of malaria. One care-giver (0.4 %) admitted that it was due to ‘too much intake of oily food’ while 5 (1.8 %) did not know the cause of malaria. Majority of the respondents, 249 (92.2 %) correctly identified ‘hotness of the body or fever’ as the most common sign and symptom of malaria. Other signs and symptoms identified included: ‘generalized body weakness’ (59.3 %), ‘loss of appetite’ (47.4 %), and headache (43.0 %). On malaria prevention, one-hundred and fifty (55.6 %) admitted that ‘using mosquito nets at doors/windows’ can be a means of preventing mosquito bite and of course malaria, one hundred and thirty nine (51.5 %) of them accepted that ‘sleeping under ITN prevents malaria, while 79 (29.3 %) opted for ‘spraying insecticide every night’.

### Fever and mortality

One hundred and nine respondents (41.6 %) admitted that at least one under-5 year child under their care had fever 2 weeks prior to the index study; one hundred and fifty respondents (57.3 %) did not have any child with fever for the same period as in the above. For the question to determine incidence of under-five mortality from among the care-givers, a total of 35 under-5 deaths were recorded. Out of the 35 deaths, fourteen (40.0 %) were fever-associated, twelve (34.3 %) were non-fever associated, while for 9 (25.7 %), it could not be ascertained whether the deaths were associated with febrile illness or not.

There was significant association between those with fever and presence of parasitaemia. Those with parasitaemia either by RDT or microscopy testing were more likely to develop fever than those with no parasitaemia, (*p* < 0.001). There was no significant difference between tribe, care-givers’ age and ownership of ITNs.

The proportion of febrile patients that had parasitaemia by RDT testing was 55.4 % (46/83), while that by microscopy was 61.2 % (41/67).

The proportion of children under the age of five years that had positive parasitaemia was less among care-givers who had tertiary education (46.3 %), than among those who did not (53.7 %) (*p* > 0.05). Malaria parasitaemia increased with the age of the child; it was 15.5 % for age group 1–11 months, 38.0 % for age group 12–35 months and 46.5 % for age group 36–59 months (*p* > 0.05).

Table 3 shows the result of logistic regression of household ITNs use on parasitaemia among under-5. The predictor (independent) variables included were under-5 child-ITN usage, caregiver’s age, caregiver’s education and child’s age. The outcome (dependent) variable measured was presence or absence of parasitaemia among under-5 children that slept under any mosquito net and those that did not. Here, under-5 child sleeping under a mosquito net a night before survey, lowering child age, increasing caregivers age, and higher caregivers educational level were associated with lower odds of developing parasitaemia, though none was statistically significant. Table 3 also shows no significant association between treatment modalities for a febrile child and tribe, age or educational levels of the care-givers.

**Table 3:** Analysis of modalities of treatment, ITN use, and effect of ITN use on parasitaemia

Variables	Univariate analysis of modalities of treatment for a febrile child		Multivariate analysis of ITN use & effect of ITN use among under-5 children on parasitaemia^β^	
	*X* ^2^	*p*-value	OR (95 % CI)	*p*-value
Child age (months)	0.539	0.764		
1-11			0.40 (0.147-1.106)	0.08
12-35			0.37 (0.305-1.550)	0.37
36-59 (reference)				
Care-givers age (years)	0.559	0.756		
18-25			0.60 (0.149-2.443)	0.479
26-35			1.40 (0.492-4.001)	0.526
36 & above (reference)				
Care-givers tribe	0.131	0.937		
Igbo			1.28 (0.648-2.547)	0.473
Others			1.40 (0.623-3.107)	0.421
Efik (reference)				
Care-givers education	2.810	0.094		
Primary/Secondary			1.35 (0.644-2.811)	0.430
Tertiary (reference)				
ITN utilization			0.68 (0.322-1.419)	0.3

*OR* Odds ratio, CI Confidence interval

*X* ^2^ Chi-sqare

^β^ -2Loglikelihood = 188.19; Hosmer&Lemeshow Test = 0.99

*Significant *p* < 0.05

A statistically significant association was observed between ownership of ITNs and care-givers education (*p* < 0.05) (Table 4). No significant association was found between sleeping under an ITN and tribe, child’s age, educational levels of the care-givers (*p* > 0.05) (Table 4).

**Table 4:** Determinants of ownership and use of ITNs among respondents

Variables	Univariate analysis determinant of ITNs ownership		Multivariate analysis determinants of ITNs ownership^α^		Univariate analysis determinants of ITNs use		Multivariate analysis determinants of ITNs use^β^	
	*X* ^2^	*p*-value	OR (95 % CI)	*p*-value	*X* ^2^	*p*-value	OR (95 % CI)	*p*-value
Child age (months)	4.462	0.107			3.360	0.186		
1-11			0.43 (0.157-1.153)	0.03*			1.35 (0.594-3.089)	0.471
12-35				0.334				0.471
36-59 (reference)			0.67 (0.301-1.503)				0.78 (0.403-1.522)	
Care-givers age (years)	1.384	0.591			12.917	0.002*		
18-25			0.5 (0.140-1.808)	0.292			1.96 (0.526-7.422)	0.314
				0.432				0.020*
26-35								
36 & above (reference)			0.65 (0.227-1.886)				3.39 (1.214-9.471)	
Care-givers tribe	3.487	0.175			0.265	0.876		
Igbo			2.2 (0.9041-4.911)	0.06			1.36 (0.668-2.699)	0.375
Others				0.16				0.119
Efik (reference)			1.6 (0.827-3.221)				0.55 (0.260-1.166)	
Care-givers education	6.960	0.008*			0.587	0.441		
Primary/Secondary				0.03*				0.990
Tertiary (reference)			0.52 (0.288-0.930)				1.0 (0.546-1.846)	

*OR* odds ratio, *CI* confidence interval

*X* ^*2*^ Chi-sqare

^α^-2Loglikelihood = 287; Hosmer&Lemeshow Test = 0.307

^Β^-2Loglikelihood = 273; Hosmer&LemeshowTest = 0.728

*Significant *p* < 0.05

However, the result of the multivariate logistic regression shows that care-givers with no, primary or secondary education were less likely to have bed nets than their counterparts with tertiary education even after other determinants –age and tribe were adjusted for (*p* < 0.05; *OR* = 0.52). A similar model was fitted for possible predictors for ‘sleeping under the net’. Only care-givers age gave a statistically significant result, with younger care-givers not likely going to have under-five children that will sleep under a net (*p* > 0.05) (Table 4).

## Discussion

In this study, the prevalence of parasitaemia (by RDT) was 32.2 % while that by microscopy was 40.1 %. The proportion of febrile patients that had parasitaemia by RDT was 55.4 % while that by microscopy was 61.2 % (*p* < 0.05). This malaria prevalence of 40.1 % was higher than 12 % reported in Tanzania [14] and 6 % reported in Pakistan [15] and lower than 53.8 % reported in a relatively similar study in Nigeria [12].

Mazigo*et al*. [14], in their study found out that 52.7 % of the children that had positive parasitaemia were also febrile. In a similar study in Gabon, about 40 % of the children in a hospital who were presented with fever or history of fever also had malaria parasite-positive blood film [16]. Nigeria Malaria Indicator Survey reported a much lower proportion of febrile children who tested positive for malaria: 11 % using RDT and 12 % using microscopy than obtained in our study [12]. This result indicates that for the majority of the children, malaria parasitaemia occurred without fever whereas in this index study, more than half (61.2 %) of the children who had malaria parasitaemia also had fever.

Earlier studies had reported higher proportion of febrile patients that were parasitaemic. Ejezie and Ezedinachi [17], in their study in Calabar, found that 74.9 % of the parasitaemic subjects had high grade temperatures of 38 °C and above. Mabunda*et al*. [18] also reported that 72.4 % of the febrile children in their study were parasitaemic. Acquired protective immunity could offer an acceptable reason for presence of malaria parasitaemia without febrile illness and it has been shown to increase with age [19]. Difference in season of study could be a plausible reason for the variation in malaria prevalence [20]. Malaria prevalence, in this study, though not statistically significant, was found to increase with the age of the child regardless of the test used. This was in agreement with the findings of Nigerian Malaria Indicator Survey of 2010 [12].

### Malaria control and prevention

During the survey, caregivers were asked if they knew specific measures to prevent malaria attack. Fifty-five point six percent (55.6 %) of the caregivers mentioned using nets on doors and windows, 51.5 % mentioned sleeping under a mosquito net, while other responses were: destroying mosquito breeding places (51.1 %) and spraying insecticides every night (29.3 %). The Nigerian Malaria indicator survey of 2010 [12] reported sleeping under a mosquito net (77.2 %, South-South region), destroying mosquito breeding places (8 %), and spraying insecticides (20 %). There is no readily available answerto the 25 % decline in awareness concerning sleeping under the mosquito nets as an effective means of preventing malaria. Perhaps, the perceived rise in distribution of ITNs over the region was not accompanied by adequate information, education and communication (IEC).

### ITNs Ownership

Net ownership, as determined by possession of at least one mosquito net in a household was found to be 71.5 %. The proportion of households who had at least one mosquito net has risen far above the reported proportion of 42 % obtained in 2010 [12], 8 % in 2008 (NDHS, 2008) and 2 % in 2003 [21]. This finding was consistent with the finding of one recent study in Abuja, Nigeria [22] in which mosquito net ownership pre-and post-intervention study were 58 % and 100 % respectively. Daboer*et al*. [23] in their survey in Jos, Nigeria reported that 55.3 % of caregivers of under-5 children owned nets; a rise the authors attributed to the on-going campaigns of ITN distribution. A contrary finding was obtained in a study in Guinea [24] in which there was a decline in net ownership from 97 % in 2008 to 65 % in 2009. This sharp scale-up in bed net ownership by households in this current study is traceable to more aggressive ITN mass distribution through the support of the Global Fund, DFID, World Bank and Support for the National Malaria Control Programme, and the MDG-assisted funds in Nigeria. The nets were given free of charge most of the time, via Primary Health Centres which are usually at close proximities to households including rural dwellers.

Some variables are known to influence ownership of mosquito nets. Some of these factors have been reported to include level of education, wealth index, family size and residence among others [25]. In this study, variables like caregiver’s tribe, age and education were tested; only caregiver’s educational level was statistically significantly associated with net ownership. It can also be used as a predictor for net ownership, with care-givers with lower educational attainments more likely to own at least one mosquito net than those with higher educational status. Oresanya*et al*. [26] observed that the presence of an educated caregiver in the household raised the odds of owning a net by 42 % in the north, while this was not predictive in the south part of Nigeria after controlling other variables. Whereas the above mentioned study tested education and none education, ours, conducted also in the southern part of Nigeria examined higher and lower educational status. Contrary to our finding, a similar study in Kenyareported that higher education was associated with possession of mosquito nets. Higher education, we expected, should have the ability to better equip caregivers with necessary information about the importance of ownership and utilization of mosquito nets in malaria prevention and control. Obtaining a contrary finding in our study is indeed puzzling.

About two-third of the respondents who do not own at least a net stated that ‘nets were not available’ as a reason for not having one. For most of them, the non-availability of the nets meant they did not know where/how to get one. Other reasons given for not having mosquito net included: ‘does not like to use net’ and ‘there is no mosquito in my residence’. Misconceptions about causes of malaria and prevention modalities are also valid reasons for non-ownership and utilization of nets [27].

### ITNs Utilization

Twenty five-point four percent (25.4 %, 68/267) of children under age 5 among those interviewed slept under a net the night before the survey. Compared to previous NDHS and NMIS surveys in Nigeria, the sustained rise in net utilization was lost. The percentage of children under age five who slept under ITNs has increased steadily and substantially from 6 % in 2003 [21], to 12 % in 2008 [28] and to 26.7 % in 2010 by NMIS survey (26.7 % specifically for South-South zone where Calabar belongs, and 30.3 % generally) [12]. The decline in net utilization was also noticed among households that owned at least one ITN, only 35.6 % slept under an ITN the night before the survey as against 55.1 % in NMIS survey [12]. The rate of net use has varied over time and in different geopolitical regions in Nigeria. Oresanya*et al*. [26] in their study in Abuja reported net utilization of 11.5 %, while 37.2 % was reported in Rivers State by Tobin-West and Alex-Hart [29].

This current study showed that whereas 71.5 % caregivers with under- 5 children owned at least one ITN, only 25.4 % of the children used a net a night before the survey. The high discrepancy (46 %) between ownership and utilization of ITN in this study could not easily be explained out and as such calls for great concern. It probably shows that there is need for adequate motivation before ownership will translate to utilization. It does appear that in the last 2 years before this index study, large scale net campaigns and distribution was carried out in Calabar and since ITNs were given free of charge, caregivers were poised to have them; however, they lacked the motivation to use them. Tobin-West and Alex-Hart [29] also reported a similar finding in their study where only one-third of those that owned nets, slept under a net the night before survey. In another study, out of the 55.3 % caregivers with under- 5 children that owned ITNs, only 40 % utilization was recorded a night before study.

The most common reason given among the caregivers; that had at least one ITN for not using it was that the weather was too hot (77.2 %). This same reason has also been reported in other similar studies and has been attributed to the hot tropical climate of the sub-Saharan African region [12, 26, 30, 31]. Other reasons were: having difficulties hanging it, and that there were no mosquitoes around their residence and these findings were consistent with an earlier study [12].

Higher educational levels in previous studies [22, 26] have been associated with appropriate net usage. In our study, possession of higher education was not statistically associated with net utilization (*p* > 0.05). Demographic characteristics like child age, care-givers’ age, education, and ethnicity have been known as possible predictors of net use in other studies [26]. In this study, only care-givers age was established as predictor (*p* < 0.05). Our study showed that older care-givers are more likely to have their children sleep under net than younger care-givers. This finding could have been as a result of past experience these older care-givers have had with caring and parenthood.

### Impact of ITNs on the under-five children

Assessment of the impact of ITN coverage and/or utilization on health outcomes is usually difficult. This is usually due to poor routine health information and vital registration systems; making determination of malaria-specific mortality and morbidity almost impossible [32]. Few studies that have attempted analysing the impact of mosquito net ownership and usage on children have used different approaches [33, 34]. In this study, the relationship between household use of ITNs among under-5 children and malaria parasitaemia was analysed using logistic regression analysis. We observed a 32 % reduction in malaria parasitaemia among under-5 net users which was not statistically significant. Lack of statistical significance may have stemmed from the small sample size involved. Previous studies by Lim et al. [32] reported a pooled relative of 24 % reductionin parasitaemia prevalence in children while Lengeler [35] reported a 50 % reduction in clinical episodes and malaria parasitaemia.

### Care-givers knowledge of malaria and treatment decision to under-5 febrile child

Malaria prevention and control measures aim at preventing mortality and reducing morbidity and also malaria-associated economic losses. Lack of knowledge about malaria and its mode of transmission will hamper appropriate preventive measures. In our study, care-givers were asked questions to ascertain their knowledge of causes, signs and symptoms, and means of prevention of malaria. Almost all the caregivers (97.8 %) identified mosquito bite as the cause of malaria. This finding was consistent with those of previous studies [12, 36] but higher than the finding by Oreagba*et al*. [37]. This awareness is a good one and could have contributed to the high level of ownership of mosquito nets among care-givers observed in this study. Most of the care-givers also identified fever as the commonest symptom of malaria. This finding was in agreement with the findings of previous studies [38, 39]. The recognition of fever by the majority of the caregivers as a symptom of malaria is a welcome development because early treatment depends on prompt recognition of symptoms and signs of malaria in the household [40]. A worrisome finding was that only 4.1 % of the caregivers acknowledged that disorientation/incoherent speech, which occurs in severe malaria, was a complication of malaria. The implication of this finding is that most caregivers would exclude malaria much the same way they behave when their children have febrile convulsion and might resort to other means of intervention like going to the Traditional healer [41]. The association between ‘incoherent speech’ and severe childhood malaria should be highlighted and incorporated into health education and health promotion programmes. This will correct anomalies in care-givers’ treatment seeking behaviour. Ability of the caregivers to recognize danger signs of malaria is an important factor for early home management or for seeking treatment at health facility [42].

Treatment seeking behaviour among caregivers has been shown to be related to the cost, availability and cultural beliefs about the causes and effective cures for malaria-like symptoms [43]. Among the caregivers who had under-5 children with febrile illness two weeks prior to this survey, 47.7 % sought for treatment first at government hospital nearby. This finding was low compared to reported value of 65.6 % in a previous study in Nigeria [23] and 71.5 % in a study in Ethiopia [36]. The finding in this study that 28.4 % of the caregivers would resort to self-treatment at home was fairly high compared to 1.4 % found in Ethiopia [36] and 3 % in Nigeria. Only few caregivers (10.1 %) resorted first to Chemist/Patent medicine vendors compared to 37 % [37] and 57.4 % [12] reported by previous studies. Unlike other previous similar studies in Nigeria [44, 45], where traditional/herbal homes were among preferred health facilities care-givers sought after, none of the care-givers in this study accepted ever going to the herbalist for treatment of their febrile children. Perhaps, variation in study areas between the previous studies which were carried out in rural areas and this current study carried out in the metropolitan town of Calabar, Nigeria could explain the difference. The preferred choice of the care-givers to seek treatment first in government hospital for their febrile children may not be unconnected with the high literacy level of the respondents who probably knew they would get better care delivery from such centres. The cost of health care delivery has been one of foremost determinants of treatment seeking behaviour of care-givers [46]. The free medical services in government hospital in Ethiopia could have contributed to higher proportion of health seekers that used government hospitals there, than it was found in this study. The high number of under-5 care-givers that indulged in self-treatment of their febrile children at home in this study highlights the need for Health extension workers to educate care-givers on home-based management of malaria. Such enlightenment programmes should include recommended anti-malarial drugs and dosages and to be able to detect signs and symptoms of severe malaria that may demand expertise management.

### Limitations of the study

Our study has some limitations. First, we tried to replicate a Nigerian Malaria Indicator Survey (NMIS) of 2010 [12], however we believed that the findings would not very much compare with NMIS; in that, ours was facility-based and the tools used were different. We observed that our sample size was small, and thought this could have been responsible for the study’s lack of power to detect many significant relationships from our data. The tool used in our research (for example questionnaire) encouraged “self-reported data”, not allowing for independent verification. Self-reported data has many sources of potential bias we considered as limitations such as selective memory and exaggeration. Finally, the sampling technique we used (convenience sampling) helped us to have easy access to the study participants in good time, however it could have introduced sampling bias, not allowing for good representation of the entire population.

## Conclusions

A parasitaemia prevalence of 40.1 % obtained in this study can still be seen to be high considering recent scale up in malaria prevention campaigns in the area. Fever was significantly associated with malaria parasitaemia. This means a lot of febrile illnesses among the under-five children in this area might still be due to malaria infection. Respondents identified various methods that are used to prevent/control malaria infection, with majority acknowledging putting net at windows and doors, followed by sleeping under mosquito nets and the use of insecticide sprays. Household ownership of nets was very high compared to many recent studies, however, the net ownership did not translate to use as there was much discrepancy between ownership and usage of the net. There was no statistically significant reduction in malaria parasitaemia with the use of mosquito nets over non-use among the under five children studied, an effect that could have arisen due to smallness of sample size. The respondents demonstrated good knowledge of the cause and symptoms of uncomplicated malaria, however, only few knew the signs and symptoms of severe malaria. Majority of the respondents also demonstrated the deadliness of malaria among the under five children via their treatment seeking behaviours. Most of them would prefer to take their children to government hospital first, possibly hoping to obtain best care delivery there. Fairly good number of care-givers would rather prefer to ‘try their luck’ by giving self-medication at home first.

## Additional file

Additional file 1: Questionnaire. (DOCX 18 kb)

## Abbreviations

ACT: artemisinin-combination therapy
CI: confidence interval
DFID: department for international development
IEC: information, education and communication
IMCI: integrated management of childhood illness
IRD: indoor residual spraying
ITN: insecticide-treated net
ITNs: insecticide-treated net
LLINs: long lasting insecticidal nets
MDG: millennium develoment goal
NDHS: Nigeria demographic and health survey
NMCP: national malaria control programme
NMIS: Nigerian malaria indicator survey
NPC: national population commission (NPC)
OR: odd ratio
RDT: rapid diagnostic test
SPSS: statistical package for social sciences
