# Supplementary material for: Malaria Parasitaemia and the use of insecticide-treated nets (INTs) for malaria control amongst under-5 year old children in Calabar, Nigeria
Source: BMC Infect Dis. 2016 Apr 14;16:151. doi: 10.1186/s12879-016-1459-5 (PMC4832572; doi:10.1186/s12879-016-1459-5)
Supplement: Additional file 1: — Questionnaire. (DOCX 18 kb) [file 12879_2016_1459_MOESM1_ESM.docx]

**QUESTIONNAIRE**

**Introduction**

Greetings! My name is _____________________________ and I am a Post-graduate student of Medical Microbiology student of Olabisi Onabanjo University, Ogun State. We are conducting a survey that asks women and men various questions about causes, symptoms, prevention and treatment of Malaria in their household. This study has been reviewed and granted approval by the Research and Ethics Committee of the University of Calabar Teaching Hospital, Calabar for the study period of November, 2012 - December, 2013. We would very much appreciate your participation in this survey. This information will help the government to plan health services. The survey usually takes between 5 and 10 minutes to complete. Whatever information you provide will be kept strictly confidential and will not be shown to other persons. Should you have any questions, feel free to call or contact the person below:

**Anthony Iwuafor:** Principal Investigator: **Email** [achiton@yahoo.com](mailto:achiton@yahoo.com); **Phone**: 08033441539

Participation in this survey is voluntary, and if we should come to any question you don’t know, just say so and we will go on to the next question; or you can stop the interview at any time. However, we hope that you will participate in this survey since your views are important.

At this time, do you want to ask me anything about the survey? May I begin the interview now?

**ON THE CARE-GIVERS:**

1. How old were you at your last birthday? Age in years __________
2. Have you ever attended school? Yes [ ] No [ ]
3. What is the highest level of school you attended: Primary [ ] Secondary [ ]

Tertiary [ ]

1. What is your ethnic group? Efik [ ] Hausa [ ] Igbo [ ] Yoruba [ ]

Other (specify)______________

1. What do you do for a living? Civil servant [ ] other (specify)_____________

**ON THE CHILDREN:**

1. How many children do you have? ____________
2. Have you ever born a child who was alive and later died? Yes [ ] No [ ]
3. If yes to question No.7, how many under 5-year old have died? ___________
4. Do you think any of the deaths in # 8 was due to fever-related condition? Yes [ ]

No [ ]

Not sure [ ]

1. Do you always complete your children immunization as/at when due? Yes [ ]

No [ ]

Don’t know [ ]

1. In the last 2 weeks has any of your under-five year old been sick with fever at any time? Yes [ ]

No [ ]

Don’t know [ ]

1. If yes to question No. 11, where did the child first seek treatment?

Government hospital [ ] Government health centre [ ]

Private hospital [ ] Pharmacy [ ]

Chemist Shop [ ] Traditional Practitioner [ ]

Drug Hawker [ ] Self-treatment at home [ ]

Other [ ] Don’t know [ ]

1. How much did the treatment cost? Include cost of consultation, drugs, tests: ______

**MALARIA-RELATED QUESTIONS**

1. At any time in the past 12 months, has anyone come into your dwelling to spray the interior walls against mosquitoes? Yes [ ]

No [ ]

Don’t know [ ]

1. If yes to question No.14, who sprayed the dwelling?

Government worker/Programme [ ]

Private company [ ]

Other (specify) _________________

1. Does your household have any mosquito nets that can be used while sleeping?

Yes [ ]

No [ ]

1. If No to question No.16, why doesn’t your household have any mosquito nets?

No mosquitoes [ ]

Nets not available [ ]

Don’t like to use nets [ ]

Nets too expensive [ ]

Other (specify) ______________

1. If yes to question No.16, how many mosquito nets does your household have? _____
2. How many months ago did your household obtain the mosquito net?

< one month [ ]2 – 12 Months [ ]

13 - 24 moths [ ] >24 months [ ]

1. Where did you obtain the nets?

Net distribution campaign [ ] Primary Health Centre [ ]

Government Hospital [ ] Private hospital [ ]

NGO/Mission clinic [ ] Church/Mosque [ ]

Pharmacy [ ] Patent Medicine store [ ]

Shop/Super market [ ] Hawker [ ]

Don’t know [ ]

1. Did you buy the net or was it given to you free?

Bought [ ]

Free [ ]

Don’t know [ ]

1. When you got the net, was it already factory-treated with an insecticide to kill or repel mosquitos? Yes [ ]

No [ ]

Not sure [ ]

1. Since you got the mosquito net, was it ever soaked or dipped in a liquid to kill or repel mosquitos? Yes [ ]

No [ ]

Not sure [ ]

1. Did anyone sleep under this mosquito net last night? Yes [ ]

No [ ]

Not sure [ ]

1. If No to question No.24, why didn’t anyone sleep under the net? (Choose one please)

No mosquitoes [ ] No malaria [ ]

Too hot [ ] Difficult to hang [ ]

Don’t like smell [ ] Feel ‘closed in’ or constricted [ ]

Net too old or torn [ ] Net too dirty [ ]

ITN provokes coughing [ ] Don’t know [ ]

Other (specify) ____________

1. What do you think is the cause of malaria? (choose one please)

Mosquito bite [ ] Staying in the sun too long [ ]

Too much stress/hard work [ ] Too much intake of alcohol [ ]

Juju/witchcraft [ ] Too much intake of oily foods [ ]

1. What do you think is/are signs/symptoms of malaria? (Multiple choice allowed)

Hotness of the body/fever [ ] Joint pains [ ]

Headache [ ] Loss of appetite [ ]

Vomiting [ ] Generalized body weakness [ ]

Talking nonsense/disorientation [ ] Body pains

Dark coloured urine [ ] Other (specify) ____________

1. How do you think malaria can be prevented? (Multiple choice allowed)

Using mosquito nets on doors and windows [ ] Keeping out of sun [ ]

Using mosquito coils [ ] Spraying insecticide every night [ ]

Using mosquito repellent cream [ ] Cleaning bushes around the house [ ]

Taking malaria tablets weekly/monthly [ ]

Sleeping under Insecticide treated nets [ ]

Destroying mosquito breeding places [ ]

Don’t know [ ]

Other (specify) _______________

Result of Malaria test: Positive [ ] Negative [ ]

Temperature: Fever Yes [ ] ______ºC Fever No [ ] ______ºC
